# Concepts for drone based pipeline leak detection

**DOI:** 10.3389/frobt.2024.1426206

**Published:** 2024-08-15

**Authors:** Lutz Bretschneider, Sven Bollmann, Deborah Houssin-Agbomson, Jacob Shaw, Neil Howes, Linh Nguyen, Rod Robinson, Jon Helmore, Michael Lichtenstern, Javis Nwaboh, Andrea Pogany, Volker Ebert, Astrid Lampert

**Affiliations:** ^1^ TU Braunschweig, Institute of Flight Guidance, Braunschweig, Germany; ^2^ Air Liquide, Campus Innovation Paris, Paris, France; ^3^ National Physical Laboratory (NPL), Teddington, United Kingdom; ^4^ Institut für Physik der Atmosphäre, Deutsches Zentrum für Luft- und Raumfahrt, Oberpfaffenhofen, Germany; ^5^ Physikalisch-Technische Bundesanstalt (PTB), Braunschweig, Germany

**Keywords:** drone, leak detection, methane, pipeline, SDG13, plume modeling, release experiment

## Abstract

The quickly developing drone technology can be used efficiently in the field of pipeline leak detection. The aim of this article is to provide drone mission concepts for detecting releases from pipelines. It provides an overview of the current applications of natural gas pipeline surveys, it considers environmental conditions by plume modelling, it discusses suitable commercially available sensors, and develops concepts for routine monitoring of pipelines and short term missions for localising and identifying a known leakage. Suitable platforms depend on the particular mission and requirements concerning sensors and legislation. As an illustration, a feasibility study during a release experiment is introduced. The main challenge of this study was the variability of wind direction on a time scale of minutes, which produces considerable differences to the plume simulations. Nevertheless, the leakage rates derived from the observations are in the same order of magnitude as the emission rates. Finally the results from the modeling, the release experiment and possible drone scenarios are combined and requirements for future application derived.

## 1 Introduction

In order to mitigate global warming, the reduction of the greenhouse gas methane with a mean atmospheric lifetime of 11.8 years and a greenhouse warming potential of 81.2 over 20 years compared to ${CO}_{2}$ is essential (Intergovernmental Panel on Climate Change (IPCC), 2023; Smith et al., 2021). With increased use of natural gas, also methane emissions are increasing (Wang et al., 2022). Emissions vary strongly between countries, which depends on infrastructure and handling (Chen et al., 2023). According to the Global Methane Tracker (IEA, 2023) natural gas accounted for 27.5% of energy sector methane emissions in Europe in 2022, and reductions are of major importance for the European energy transition (Shirizadeh et al., 2023). One source of methane is leakages from pipelines. Current estimations of annual methane emissions from pipeline leaks in the US are in the range of 1.25–2.66 $\cdot \;10^{6}$ t (McVay, 2023), and it is estimated that more than 630,000 leaks occur in distribution pipelines in the U.S. (Weller et al., 2020). The aim is to reduce methane pipeline leakages in the EU by 29% compared to 2005 levels until 2030 (European Union Agency for the Cooperation of Energy Regulators (ACER), 2023). Further, leakage detection is in the interest of owners and operators worldwide for economic reasons and to reduce risks (Benyeogor et al., 2020; Dutzik et al., 2022).

There are different types of pipelines: *Gathering*, *transmission* and *distribution pipelines*. *Gathering pipelines* are used for transporting raw gas from production to processing sites above or below the surface. They typically consist of pipeline segments made of plastic, steel or iron of up to 100 km length, pipeline inspection gauges for maintenance, and valves to stop and direct gas flow. The typical pressure is in the range of 55 bar (Roscioli et al., 2015).

There are roughly 200,000 km of *transmission pipelines* in the EU (European Union Agency for the Cooperation of Energy Regulators (ACER), 2023). They have a typical length of different parts of 100 km, high pressure up to 85 bar, and they are located typically between 1 m and 2 m below the surface in open areas like fields and grass land. According to the German Technical and Scientific Association for Gas and Water (DVGW), a detection limit of 2.5 L ${min}^{-1}$ is required for leak detection (DVGW G 501 method) (DVGW, 2012). Leaks can range from very small to complete loss of integrity, e.g., the NordStream pipelines (Harris et al., 2023). Operators have no tolerance for leaks of any size during transmission because of the risk that leaks can grow in size very rapidly and become major hazards and risks to national energy supplies. In terms of upper limit within the scope of interest of this paper, the leak size can be up to around a few percentage of the total flow through the pipeline, where flow or pressure metering between terminals and compressor stations may start to recognise an issue. Typical flows through some of the larger transmission pipelines may be on order of 10 million cubic metres per day, so a few percentage of that flow may be on the order of 1 $\cdot 10^{5}$ L ${min}^{-1}$ . However, at this flow and pressure the gas will likely start to self-excavate the pipeline and it would become easily identifiable by public or a routine visual network inspection.

There are more than 2 million km of *distribution pipelines* and over 20,000 compressor and pressure reduction stations in the EU (European Union Agency for the Cooperation of Energy Regulators (ACER), 2023). They provide gas to individual households, and they are located under streets of settlements. They have a typical diameter of up to 30 cm, but there is a lot of variability between countries, e.g., it can be up to 24 inch (61 cm) diameter in the United States (Pipeline Safety Trust, 2015). Common big leaks are in the size of 15–50 L ${min}^{-1}$ (Ulrich et al., 2019). A recent leak from a gas distribution pipe in the United Kingdom was estimated to be ∼ 15,000 L ${min}^{-1}$ (Dowd et al., 2023). Leaks are categorized according to safety, taking into account not only the emission rate, but also potential underground accumulation and the proximity to infrastructure (Maazallahi et al., 2023).

The worldwide combined pipeline length was around 1.2 × 10^6^ km in December 2022 (Global Energy Monitor, 2023).

There is a need for automated continuous monitoring systems for leak detection (Golston et al., 2018). Monitoring and leak detection have been done traditionally by ground-based measurements in walking or driving surveys, and airborne surveys. For example, the United Kingdom transmission network has a piloted helicopter based visual survey on a fortnightly basis. DVGW-G501 is active in Germany through services offered by companies, e.g. Adlares et al. (2023). Leaks from distribution networks are commonly reported by the public in the United Kingdom as the gas is odourised at the distribution level.

Recent technological advances in the fields of drones and methane sensors, in particular the availability of small-size and low-weight methane sensors, provide the potential of drone-based leak detection. However, drones are currently mainly deployed to complement and not yet substitute classical technologies of mobile leak detection (Ravikumar et al., 2019). Drone-based leak detection has the potential to be more flexible, more efficient, maybe cheaper and also safer compared to the classical methods, as no person is required to approach the leakage spot. Drones are capable to fill the gap of atmospheric monitoring in the altitude range of 0–1,000 m with high resolution measurements in the range of few seconds and meters. Optimization methods for determining a suitable flight path in pipeline networks for inspection and leakage detection have been discussed (Yan et al., 2019). Drone measurements in the area of a known leakage have been performed in Poland, demonstrating that low flight altitudes should be chosen for sensors that average methane concentrations over the column between surface and measurement altitude (Iwaszenko et al., 2021). However, the application of drones strongly depends on the requirements, ambient conditions, regulations governing drone flights, and the mission design.

A review about drones for quantifying methane emissions (Shaw et al., 2021) showed the advantage of high spatial flexibility, and limitations associated with the typically high atmospheric variability, and accuracy of current instruments. Comparing the estimation of emission strength, drone-based measurements had generally larger errors than other techniques (Liu et al., 2023). A more general review about leak detection and quantification presents different sensors and methods for determining emissions, showing the advantages and disadvantages of the systems (Hollenbeck et al., 2021), and in particular the applicability for drone measurements: Advanced leak detection and quantification mostly combine a numerical model, e.g., the Gaussian plume dispersion, to take into account atmospheric effects and variability, and mobile measurements on different scales. The value of measurements strongly depends on different parameters, like operator skills, cost of equipment, setup time, survey time, and the required precision and accuracy.

Besides leakages from rural pipelines, there may be other sources of methane emissions from e.g., agriculture (Jackson et al., 2020) or waste treatment and landfills (Bogner et al., 1995; Bogner and Matthews, 2003), which may be separated by measuring additional tracer gases (Kille et al., 2019). The emissions from the different sources might mix and could then trigger false alarm for automated systems, which has to be taken into account.

In an underground release experiment, a sharp gradient of the methane concentration was observed within 10 cm altitude above the surface and within 3 m horizontal distance of the source, emphasizing the need of accurate sensor placement and a minimum detection limit of 10 ppm for the sensors (Ulrich et al., 2019). Drone based pipeline leak detection has been done with a remote sensing device, covering each pipeline segment twice (Smith et al., 2016; Li et al., 2020). Here, the criteria to identify a leak were:

• a gradual increase and subsequent decrease of the methane concentration

• a concentration above a certain threshold value, e.g., set to 10 times the background concentration

• the same location for the flight back

For some case studies of real pipeline leakage investigations, low flight altitudes were most useful to locate leakages (Iwaszenko et al., 2021), while others use higher flight altitudes in the range of 40 m (Li et al., 2020). Automated mapping for drone based leak detection has been applied based on rectangular scan patterns with coarse and then fine resolution (Golston et al., 2018). Appropriate post-processing of the data is of high importance for identifying artificial leakages and natural sources (Barchyn et al., 2019; Ravikumar et al., 2019), including filtering for outliers due to highly or not reflecting surfaces, and, e.g., clustering the enhanced concentrations by means of machine learning (Iwaszenko et al., 2021).

For dedicated methane release experiments it is possible to measure the *in situ* methane concentration by deploying the inlet on the drone and linking it to the analyzer on ground (Shah et al., 2019), which is, however, not practical for the search of unknown leaks along a pipeline.

This article provides principal concepts for drone-based leak detection in Europe, however, it does not aim for emission rate quantification. It takes into account metrology requirements and environmental conditions (Sect. 2), and discusses different drone configurations (Sect. 3) and available methane sensors that fit in the weight, dimension and power constraints of drones (Sect. 4). Two concepts for drone-based pipeline leak monitoring are presented in Sect. 5, taking into account current legal requirements in Europe. An own example of drone-based methane measurements during a controlled release experiment in Poland illustrates the influence of wind direction variability (Sect. 6). Based on this, three scenarios of potential applications are presented in Sect. 7. Finally, the conclusions are provided in Sect. 8.

## 2 Metrology requirements for pipeline leak detection

For making use of drone-based leak detection it is an advantage to know the estimated distribution of methane for different environmental conditions. Sect. 2.1 describes the typical environmental conditions for which leak detection methods are developed in the following. Two different theoretical approaches of Gaussian distribution are used to describe suitable flight patterns for an *in situ* point sensor (Sect. 2.2), and for a path-integrating sensor (Sect. 2.3). Throughout the article, concentrations are provided in ppm, referring to volumetric concentrations. For simplicity, flow rates are given in L ${min}^{-1}$ , which refers to the normalized volumetric flow at standard conditions (273.15 K, 1,013.25 hPa).

### 2.1 Environmental conditions for pipeline leak detection

For drone-based methane detection, environmental conditions have to be taken into account (Ali et al., 2020). For remote sensing devices based on laser absorption, typically a total reflection of the ground, turbulent atmospheric conditions, which means vertical mixing, and Gaussian dispersion of the plume are assumed (Li et al., 2020). Other flux methods can be used when the plume is non-Gaussian in shape, or assumed not to be.

However, already for relatively low wind speed exceeding 4.5 m $s^{-1}$ at a leak rate of 5 L ${min}^{-1}$ , it turned out that methane leaks could not be found in release experiments and from pipelines due to fast dilution (Li et al., 2020). Therefore, release experiments have been developed which deploy dual tracers and dual transects for measuring the dispersion directly (Roscioli et al., 2015). For underground release experiments, even wind speed exceeding 2 m $s^{-1}$ led to substantial dilution, making it possible to miss the leak (Ulrich et al., 2019). On the other hand, small wind speed was found to be unfavourable for determining emission rates if a Gaussian model distribution is included in the calculations (Liu et al., 2023; Förster et al., 2024). For a fixed-wing drone with *in situ* measurement technique and a certain minimum flight altitude of 40–50 m, downstream distances of 750 m–2000 m were found to be necessary to make sure that the dispersion has evolved sufficiently for not missing the plume (Barchyn et al., 2019).

While focusing on smaller leaks with a correlated increase in costs with size less than on huge hazardous leaks the leak investigations are less time critical and weather dependent. For that reason the concepts described within the paper focus on conditions of release rates normally between 2.5 and 500 L ${min}^{-1}$ and low wind speed conditions in the range of 1.5–4.5 m $s^{-1}$ .

### 2.2 Estimated methane concentration and distribution for point measurements

The model Ventjet (Miller et al., 2021) is used in the following for the characterization of the methane plume induced by a release from a pipeline above ground and for *in situ* point concentration measurements. The expected plume properties are necessary to define the main requirements for drone-based pipeline leak detection in terms of drone flight capabilities and detection thresholds. Thus the methane dispersion was studied taking into account several conditions of release (initial methane pressure, leak diameter) and weather (e.g., wind speed, atmospheric stability).

The following conditions were studied, motivated by the own release experiment described in Sect. 6.

• Weather conditions commonly investigated in hazard studies considering atmospheric stability classes according to Gifford (Gifford, 1976)
  - 3F: wind speed at 3 m $s^{-1}$ , slightly stable atmospheric conditions
  - 5D: wind speed at 5 m $s^{-1}$ , neutral atmospheric conditions
• Additionally studied weather conditions
  - 1F: wind speed at 1 m $s^{-1}$ , slightly stable atmospheric conditions
• Several methane concentration targets from 1 ppm to 15%
• Methane pressure in the pipeline from 2 bar to 85 bar
• Leak hole diameters:
  − 0.35 mm for very small leak in case of tightness default, leaking connection
  − 4 mm for intermediate leak
  − 12 mm for small breach potentially caused by construction defect, material defect, or corrosion
  −70 mm for medium breach due to third party works for instance
• Methane release flow rates:
  - from 2.4 L ${min}^{-1}$ to 4.2 $\cdot \;10^{7}$ L ${min}^{-1}$ (corresponding to 30 mg $s^{-1}$ to 50 kg $s^{-1}$ )
  - with a specific focus on the range from 25 to 500 L ${min}^{-1}$

• Release location: at 1 m and 7.2 m altitude above ground

Pipeline full-bore rupture - which leads to catastrophic consequences - was not considered because these preliminary calculations were focused on the definition of the smallest methane concentrations to be detected by the system.

Ventjet, an engineering model dedicated to atmospheric dispersion of various gases, which provides some significant enhancements compared to the dispersion model PHAST (DNV-GL, 2014), was used to define methane plume characteristics considering the parameters described above. Indeed, Ventjet takes into account various weather condition profiles, namely, neutral D and stable F, wind speed and orientation, ground effect.

Preliminary calculations were carried out based on this approach. The main parameters considered are: an upward vertical release orientation and a surface roughness coefficient of 0.18, which is an intermediate value, recommended for an industrial environment.

**FIGURE 1:**
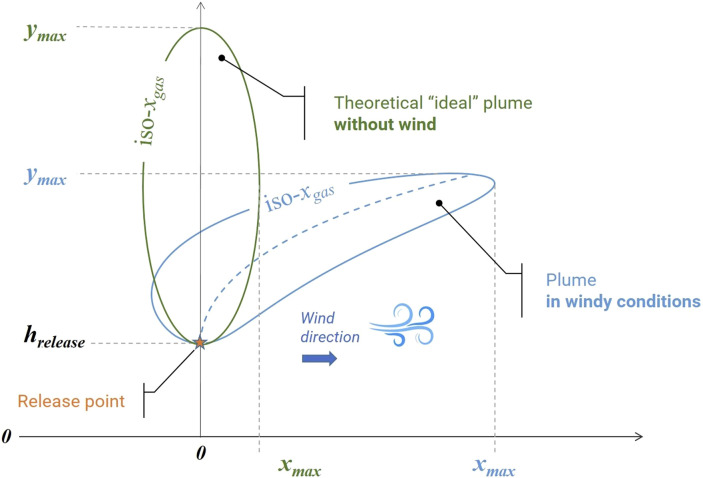
Theoretical plume shape for a buoyant gas depending on different wind conditions.

Among the calculations performed, Table 1 presents horizontal and vertical distances - respectively represented by $x_{\max}$ and $y_{\max}$ in Figure 1 - calculated in a wide range of targeted ${CH}_{4}$ concentrations (from safety (%- ${CH}_{4}$ ) to environmental (ppm- ${CH}_{4}$ ) considerations) for the smallest and the highest considered release flow rates, and for wind speeds of 2 and 5 m $s^{-1}$ .

**TABLE 1:** Calculated horizontal distance ( $x_{\max}$ ) from the release point and vertical distance ( $y_{\max}$ ) from the ground for targeted ${CH}_{4}$ concentration from 15% to 1 ppm, for the extreme release flow rates, considering different conditions of atmosphere stability, and wind speed at 2 and 5 m $s^{-1}$ . The results are only the result of the source and clean air and do not include the background concentration of approx. 2 ppm.

Targeted ${CH}_{4}$ Concentration	0.35 mm diameter—2 bar 2.4 L ${min}^{-1}$						70 mm diameter—85 bar 4.2 ⋅ $10^{7}$ L ${min}^{-1}$			
	2F		2D		5D		2F		5D	
	x	y	x	y	x	y	x	y	x	y
	[m]	[m]	[m]	[m]	[m]	[m]	[m]	[m]	[m]	[m]
15%	<0.1	<1	<0.1	<1	<0.1	<1	1	15	1.2	14
10%	<0.1	1	<0.1	1	<0.1	<1	1.6	22	2.3	20
5%	<0.1	1.1	<0.1	<1.1	<0.1	1	5	41	7	32
1%	<0.1	1.2	<0.1	1.1	<0.1	1.1	44	84	49	60
0.1%	0.5	1.3	0.5	1.3	0.5	1.2	621	157	466	114
100 ppm	2.5	1.6	2.5	1.5	1.9	1.3	5,076	290	2919	205
10 ppm	7.9	2.1	7.2	2	5.1	1.6	42,766	597	12,291	440
1 ppm	34	3.3	27	3.2	16	2.5	326,398	911	44,835	1,300

As a result, for the smallest studied leak - i.e. 2.4 L ${min}^{-1}$ (0.35 mm and 2 bar) - 10 ppm of methane could be measurable at a horizontal distance of 5–8 m from the release point (depending on weather conditions), and vertically at a distance of around 2 m above ground. Logically, these distances increase with pipeline pressure and leak hole diameter (more than a few hundred meters for the highest leaks), and higher concentrations are measured at lower distances.

Weather conditions and wind speed have a significant impact on methane dispersion and the shape of the plume (see Table 1). Thus without wind the plume will be vertically-oriented, not very wide but very high, compared to a windy weather which will induce a horizontal inclination of the plume leading to a horizontal ${CH}_{4}$ dispersion far from the release point, as illustrated in Figure 1.

The inclination and shape of the plume will depend on the release flow rate as well. In addition to the sensitivity of the embedded sensor for methane detection, these conditions will have to be considered for the flight pattern of the drone and thus achieve fine localization of the release point on the pipeline.

In this way, complementary calculations were carried out considering the flight altitude of the drone for the tests with a tighter release flow rates range from 25 L ${min}^{-1}$ to 500 L ${min}^{-1}$ . The release point is considered to be at 7.2 m height above ground, as in the experiment in Sect. 6.

**FIGURE 2:**
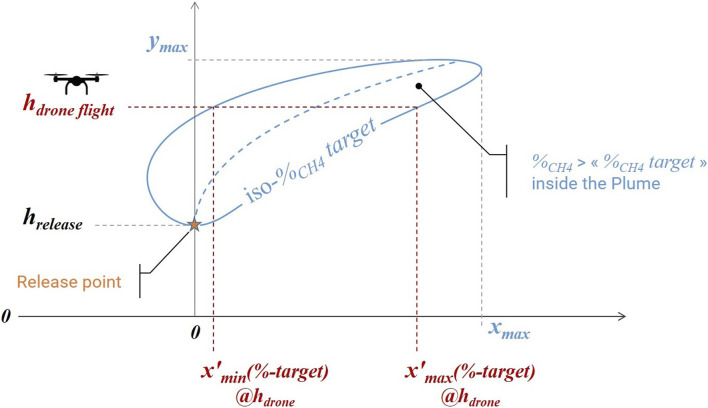
Horizontal minimum and maximum calculated distances for a targeted ${CH}_{4}$ concentration at drone flight altitude ( $h_{\mathrm{droneflight}}$ ).

For easier understanding, the considered parameters and calculated values in Table 2 are represented in Figure 2.

**TABLE 2:** Horizontal minimum and maximum calculated distances from the release point for a targeted ${CH}_{4}$ concentration at drone flight altitude, considering the release point at 7.2 m height.

Release Rate [L ${min}^{-1}$ ]	Altitude drone [m]	Wind Speed [m $s^{-1}$ ]	1 ppm ${CH}_{4}$	
			${x^{\prime }}_{\min}$ at $h_{\mathrm{drone}}$ [m]	${x^{\prime }}_{\max}$ at $h_{\mathrm{drone}}$ [m]
500	20	1.5	37	870
500	20	1	20	1,120
100	20	1	80	270
50	20	2	-	-
50	20	1	-	-
25	15	1	70	97

${x^{\prime }}_{\min}$ and ${x^{\prime }}_{\max}$ represent minimum and maximum horizontal distances from the release point where the ${CH}_{4}$ targeted concentration can be measured in the axis of the ${CH}_{4}$ plume, at the altitude of flight of the drone ( $h_{\mathrm{drone flight}}$ ). Between these two distances, the methane concentration is higher than the ${CH}_{4}$ targeted concentration. Inside the plume delimited by the iso- ${CH}_{4}$ target concentration, the measured %- ${CH}_{4}$ concentration is higher than the ${CH}_{4}$ targeted concentration as well. In other words, in the axis of ${CH}_{4}$ plume, at drone flight altitude, the following concentrations are expected.

• if distance < ${x^{\prime }}_{\min}$ , %- ${CH}_{4}\;<$ ${CH}_{4}$ targeted concentration,
• if ${x^{\prime }}_{\min}\;<$ distance < ${x^{\prime }}_{\max}$ , %- ${CH}_{4}\;>$ ${CH}_{4}$ targeted concentration,
• if distance > ${x^{\prime }}_{\max}$ , %- ${CH}_{4}\;<$ ${CH}_{4}$ targeted concentration.

Table 2 gives calculated ${x^{\prime }}_{\min}$ and ${x^{\prime }}_{\max}$ for a ${CH}_{4}$ target concentration of 1 ppm for several release rates and wind speeds.

At 20 m altitude, 1 ppm of ${CH}_{4}$ could be measured by a sensitive enough *in situ* sensor (cp. Sect. 4) at a distance smaller than 80 m from the release point for release flow rates higher than 100 L ${min}^{-1}$ . For lower release flow rates, the altitude of the flight must be decreased as shown in Table 2, since 1 ppm of ${CH}_{4}$ cannot be calculated at 20 m for 50 L ${min}^{-1}$ . Nevertheless, at 15 m altitude, for 25 L ${min}^{-1}$ , a 1 ppm ${CH}_{4}$ concentration should potentially be measurable in these conditions. Note that these values are magnitude orders, calculated from theoretical approaches. In fact, during a drone flight many parameters are variable, in particular wind speed and orientation which have a strong impact on ${CH}_{4}$ transportation and dispersion in the atmosphere.

To complete these calculations, Table 3 gives maximum ${CH}_{4}$ concentrations measurable at ${x^{\prime }}_{\min}$ and ${x^{\prime }}_{\max}$ distances from the release point at 15 m and 20 m altitude with a wind speed of 1 m $s^{-1}$ .

**TABLE 3:** Maximum ${CH}_{4}$ concentration and associated horizontal minimum and maximum distances from the release point calculated at 15 m and 20 m flight altitude, considering the release point at 7.2 m height and wind speed of 1 m $s^{-1}$ .

Flight altitude Release rate [L ${min}^{-1}$ ]	15 m			20 m		
	%-CH_4_ [ppm]	x’_*min*_ [m]	x’_*max*_ [m]	%- ${CH}_{4}$ [ppm]	${x^{\prime }}_{\min}$ [m]	${x^{\prime }}_{\max}$ [m]
500	130	19	29	20	58	106
100	6	56	96	1	80	271
50	2	59	104	0.5	97	283
25	1	70	97	0.2	154	408

The theoretically calculated values reported in Table 3 provide only indicative but valuable information for drone flight pattern definition and the required sensor sensitivity range.

### 2.3 Leak modeling with dispersion model for path-integrating measurements

To better understand dispersion properties of methane pipeline leaks, three different emission rates (2.5 L ${min}^{-1}$ , 50 L ${min}^{-1}$ , and 500 L ${min}^{-1}$ ) were modelled using the Gaussian plume dispersion software, ADMS 6 (ADMS, 2024). It should be noted that the lower end of the release rates modelled were selected to overlap with the range of expected emissions from gas pipelines based upon previous literature (Ulrich et al., 2019), and the higher release rate was adapted to the release experiment described in Sect. 6.

For all the emission scenarios modelled a point source with a diameter of 1.25 cm at a height of 7.2 m was used, these parameters were selected to try and correspond with the controlled release configuration (Table 4). For all the emission scenarios the gas composition was assumed to be pure methane. For all the simulations runs, a constant wind direction of 225° was assumed, and a range of different wind speeds was investigated (1.5 m $s^{-1}$ , 3.0 m $s^{-1}$ and 4.5 m $s^{-1}$ ), as wind speed is known to play a significant role in gas dispersion and could in turn impact upon the measurement limits of detection. Each simulation was run for a 110 m by 110 m grid (−10 m–100 m) with a resolution of 1 $m^{2}$ . The release point was located at the (x, y) coordinates of (0, 0). The model was primarily run assuming normal temperature and pressure (NTP) conditions of 293.15 K and 1,013.25 hPa as it was understood that these conditions would be representative of the conditions during the field campaign (and so it proved to be). However, some models were also run at standard temperature and pressure (STP 273.15 K and 1,013.25 Pa) for better comparison to the Ventjet model results. It should be noted that for the different conditions (i.e., NTP and STP), the volumetric flow rates were kept constant and the mass emission rates were adjusted accordingly.

**TABLE 4:** Selected input parameters for the model ADMS used within the study.

Input parameter	Unit	Value
Release diameter	m	0.0125
Release height	m	7.2
Release coordinates (x,y)		0, 0
Release rate	L ${min}^{-1}$	2.5, 50, 500
Gas composition		pure methane
Wind direction	°	225
Wind speed	m $s^{-1}$	1.5, 3.0, 4.5
Grid *x* and *y*-axes		−10 m–100 m (relative to release location of (0, 0)
Grid *z*-axis		0 m–50 m (above ground level)
Grid cell size		1 m × 1 m

The primary purpose of the dispersion modelling study was to simulate the expected results from the drone testing within this study. In this study, the drone can be considered the measurement platform. The drone utilised a handheld methane detector, LaserMethane mini, see Sect. 6.1, notably this instrument can only measure the path-integrated concentration (ppm ⋅ m) and not concentration (ppm). Conversely, ADMS typically outputs concentration values (i.e., ppm). To allow the comparison of datasets, ADMS was run between 0 m and 50 m altitude at 1 m height intervals, the path-integrated concentrations were then derived through the summation of the methane concentration output at each elevation. By doing this it was possible to determine an estimate of the path-integrated concentrations at various elevations, a summary of these maximum path-integrated concentrations observed at a height of 20 m is given in Table 5 for different scenarios.

**TABLE 5:** Maximum path-integrated concentrations and the associated (x, y) coordinates for the various emission scenarios.

Scenario no.	Condition	Wind speed [m $s^{-1}$ ]	Release rate [L ${min}^{-1}$ ]	Max. Concentration [ppm ⋅ m]	Coordinates (x, y)
1	NTP	1.5	2.5	39.9	(1, 1)
2	NTP	3.0	2.5	26.6	(1, 1)
3	NTP	4.5	2.5	19.3	(1, 1)
3	STP	4.5	2.5	20.8	(1, 1)
4	NTP	1.5	50	283.0	(2, 2)
5	NTP	3.0	50	198.0	(1, 1)
6	NTP	4.5	50	184.5	(1, 1)
6	STP	4.5	50	196.5	(1, 1)
7	NTP	1.5	500	5,147.2	(1, 1)
8	NTP	3.0	500	4543.3	(1, 1)
9	NTP	4.5	500	1276.0	(2, 2)
9	STP	4.5	500	1384.1	(2, 2)

It should be noted an assumption has been made that the point concentrations (in ppm) output from the model at 1 m separation is equivalent to the concentration path integral (ppm ⋅ m) ± 0.5 m from the point concentration measurement. To put this in simpler terms, if a concentration of 100 ppm is measured at an altitude of 5 m, it is assumed that this is equivalent to a path-integrated concentration between 4.5 m and 5.5 m of 100 ppm ⋅ m. This is thought to be a valid assumption of the data modelled but does assume a resolution of 1 m for the measurements.

As the release rate increases, the maximum path-integrated concentration also increases, as would be expected. In general, the trend maximum path-integrated concentration shows an inverse correlation with wind speed. It is also seen from Table 5 that for the equivalent volumetric flow rates at STP larger maximum concentrations were simulated. For all of the emission scenarios modelled at both conditions (NTP and STP), a maximum concentration between 6.5% and 8.5% higher was observed for the STP conditions, unsurprisingly this reflects the differences in density and, hence, the mass emission rates modelled.

**FIGURE 3:**
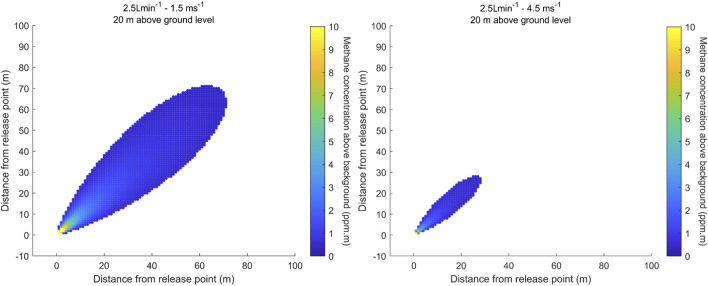
Horizontal distribution of methane path-integrated concentration above background in [ppm ⋅ m] integrated over an altitude from ground level to 20 m for a release rate of 2.5 L ${min}^{-1}$ at a wind speed of 1.5 m $s^{-1}$ (left) and 4.5 m $s^{-1}$ (right).

Modelled aerial maps of the path-integrated concentrations are used to visualise the emission scenarios: Figure 3 shows Scenario 1 and Scenario 3 at the minimum leak size of 2.5 L ${min}^{-1}$ . It illustrates the small size of the plume and the resulting requirements on the detection threshold and sampling rate, respectively airspeed.

**FIGURE 4:**
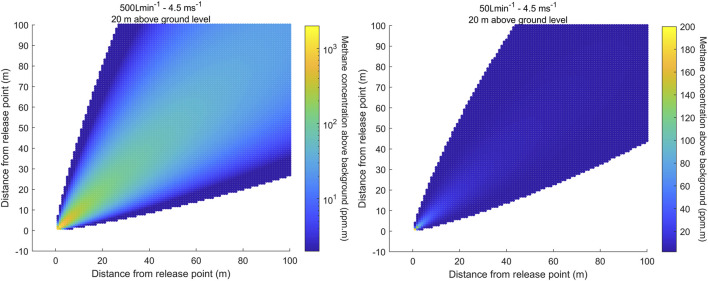
Horizontal distribution of methane path-integrated concentration above background in [ppm ⋅ m] integrated over an altitude from ground level to 20 m for a release rate of 500 L ${min}^{-1}$ (left) and 50 L ${min}^{-1}$ (right). The wind speed was 4.5 m $s^{-1}$ .

Figure 4 shows the results for a wind speed of 4.5 m $s^{-1}$ and similar conditions (NTP) during the release experiment described in Sect. 6 for Scenario 9 (emission rate of 500 L ${min}^{-1}$ , left) and Scenario 6 (emission rate of 50 L ${min}^{-1}$ , right).

### 2.4 Sensitivity requirements

The sensitivity of an instrument to be used for leak detection has to be considered in order to perform accurate and reliable measurements, as well as the measurement rate. Previous drone-based methods have published a leak sensitivity in the range of 5.8 L ${min}^{-1}$ (Li et al., 2020) but were not able to detect all leaks. A much larger emission would be more easily detectable at higher wind speed (Hollenbeck et al., 2021; Shaw et al., 2021).

Table 5 provides an overview of the required sensitivity, which strongly depends on leak rates, wind speed and drone mission parameters, like distance and altitude. For example, for low wind speed of 1 m $s^{-1}$ and a release rate of 500 L ${min}^{-1}$ , an increase of the methane concentration in the range of 20 ppm can be expected for a downwind distance between 58 m and 106 m. In contrast, for a lower release rate of 50 L ${min}^{-1}$ , a concentration exceeding 0.5 ppm can be expected for a downwind distance between 154 m and 408 m.

In terms of an efficient airborne platform for monitoring long linear assets, fixed wing drones are perhaps more appropriate than rotary wing drones, but these have minimum air speed and safe altitude requirements, meaning that sensors need to measure sufficiently frequently over the expected path length.

## 3 Drones

There is a variety of drones which may be used for leak detection, all associated with different advantages and disadvantages. The application of drones for leak detection depends on many parameters which have to be taken into account. There are relations between technical and operational requirements, which are associated with different level of cost.

Technical parameters that have to be considered include the type of drone, drone size and mass, the technical complexity and safety level of the drone, propulsion system and battery or fuel capacity. But also the sensor characteristics like size, mass, sampling rate as well as sensitivity influence the bounding and therefore the usability of the technology.

Operational parameters comprise the targeted leak size or intensity, the resulting gas distribution and concentration, the time-critical requirements, environmental conditions of operations, endurance and the required safety level.

The overall cost for drone-based leak detection is strongly influenced by the cost of the drone and sensors, size of the area to be surveyed, system development, system maintenance, crew education and training, crew size, airworthiness and approvals.

For example, to be able to fly beyond visual line of sight (BVLOS) for efficient monitoring, a higher level of robustness has to be fulfilled, including redundancy in mechanical and electric systems and control links, which results in higher cost. Another obvious dependency is the size of payload, respectively sensor. Smaller, lighter sensors only need smaller drone platforms with low air and ground impact risk in case of an accident, which means low drone costs and low regulatory costs, but also low endurance and low precision of the measurements. Both examples illustrate the interdependence between technical, operational and economical parameters.

On a first glance, fixed wing drones are more suitable for routine surveillance, leveraging the fact they can fly for long distances efficiently. Multi-rotors can be used efficiently for follow-up inspections in defined areas. In both cases appropriate sensors need to be used. For fixed wing drones a path integrating sensor with a high sampling rate is more appropriate than a point concentration detector, because the airspeed is high ( > 10 m $s^{-1}$ ). Multi-rotors could fly slower and use both types of sensors to thoroughly investigate possible emissions, taking advantage of their ability to hover and fly at very low altitudes. To regard all these aspects, the basic drone systems are introduced in the following.

### 3.1 Rotary wing drones

Rotary wing drones (multicopters) are quite common because of their simple technical structure and flight controls. The vertical takeoff and landing makes them easy to use. There are commercially available systems, like the DJI Matrice 600, which can carry several kg of payload.

The main disadvantage of using rotary drones is the need of producing the lift out of the downwash. This means they need a lot of energy even to hover. Normal flight times are between 10–30 min for electrically powered drones, which results in only small distances covered per flight and per day (Li et al., 2020). Another disadvantage for atmospheric applications is the downwash, which disturbs the atmosphere to be investigated below. This requires a careful choice of the placement of the sensor on the drone, in particular for *in situ* sensors. The optimal sensor location interacts with the drone mission: For vertically profiling the atmosphere, sensors or at least the inlet can be placed above the rotor area, and only data obtained during ascent are used for analyses (Bretschneider et al., 2022). For drones moving forward with a certain speed, a suitable sensor location can be between the two rotors oriented forward in flight direction (Smith et al., 2016). The flight strategy can be adapted to the mission. For example, investigating in a suspected point source a zigzag pattern can be flown (cp. Section 6). For routine monitoring along the pipeline, the airspeed and distance to the pipeline has to match with the sampling rate and sensitivity of the sensor and the plume width for a minimum leak size (cp. Section 2.3).

### 3.2 Fixed wing drones

Fixed wing drones have a structure that produces lift while flying through the air. Flying wings or common aircraft configurations are used to carry sensors with a propulsion system. The advantage is the lower energy consumption for flying that results in flight times of 30 min and more (Altstädter et al., 2015) depending on the type and size of fuel/battery. The disadvantage is the need of a takeoff and landing area or infrastructural support like a catapult or landing net (Bärfuss et al., 2022). With the higher minimum airspeed of fixed wing drones the flight strategy has to regard the sensor sampling rate, sensitivity and plume width as a result of the minimum leak size and weather condition. As a result normally these drones have to fly along the pipeline and more far away from the pipeline than rotary wing drones with a lower airspeed have to. For fixed wing drones a standard ratio of 1 kg payload and 5 kg system load can be assumed for first calculations. Regarding the wing size the minimum airspeed can be estimated with the lift equation.

### 3.3 Hybrid drones

Several concepts follow the principle of a hybrid fixed wing rotary drone. The disadvantages of fixed wing drones during takeoff and landing are avoided by adding rotors for vertical movement, and the negative aspect of the high energy consumption during cruise is reduced by applying the lift of a wing. The disadvantage with combining both capabilities is the lower payload mass, while requiring a higher structural mass, as well as the increasing drone complexity. Tilted wing concepts like TW-NEO (flyXdrive GmbH, Germany) or the tilted engine concepts like Wingcopter (Wingcopter, Germany) optimize the useable payload mass while increasing the complexity susceptibility. The optimization reduces the amount of engines by turning the existing engine from vertical rotation for take-off to horizontal rotation for thrust during cruise flight. Therefore, rotation mechanics for engines or wings are added and minimize the mass reduction. Furthermore, the engine-propeller-configuration has to be a compromise for the very different flight phases, respectively tasks of producing lift for low air inflow and thrust during high air inflow.

## 4 Sensors

Sensors for drone applications have to fulfil constraints concerning size, weight and power consumption. Typically, the overall payload for drone-based applications is in the range of up to several kg, depending on the drone type, dimensions, propulsion system, and drone category. High precision lightweight sensors are highly important for drone-based leak detection (Ali et al., 2020). Low-cost gas sensors, like semiconductor resistive sensors (e.g., MQ-4, (Core Electronics, 2022)) are especially small and lightweight, however, their sensitivity currently is not high enough for environmental leak detection applications (Honeycutt et al., 2019). Up until recently, the classical optical instruments which are often used in ground-based and airborne methane flux measurements, have been generally too large and heavy for drone applications (Picarro, 2022; LI-COR, 2022; Los Gatos, 2023).New solutions are provided by cheaper and more light-weight optical sensors, which can provide both the necessary detection limit and precision, as well as smaller size and weight, which makes them well suited for leak detection applications onboard drones (Schuyler and Guzman, 2017). For pipeline monitoring, real-time transmission of the ${CH}_{4}$ concentration to the operator is required, therefore, methods with sophisticated post-flight analysis of sampled air, like, e.g., the AirCore system (Andersen et al., 2018), are not taken into account here. The following sections provide a brief review of optical sensors, which have been used successfully for drone based methane leak detection. Readers are also referred to Hollenbeck et al. (2021) and Shaw et al. (2021) for more information on sensors and sensor types which have been used to measure and quantify methane emission from drones. A general review of analytical sensing and detection of methane, not specific to measurements performed on drones, is provided by Kamieniak et al. (2015). A qualitative comparison of sensor types is shown in Table 6.

**TABLE 6:** Sensor types and indicative specifications at the time of writing. Note that this is an especially generalised perspective of sensor specifications, and that individual sensor performance may exceed (or fall below) the indications presented here.

Sensor type	Point, path, area	Size	Weight	Methane precision
TDLAS (open-path)	Path	Small—medium	Low	Medium
TDLAS (cavity)	Point	Medium—large	High	High
Camera (IR, multi or hyperspectral)	Area	Small	Varied	Low
Low-cost sensor (metal oxide)	Point	Small	Low	Low

### 4.1 Remote sensing

Tunable Diode Laser Absorption Spectrometers (TDLAS) operating in the 1.65 μ m region, where strong methane absorption lines and robust diode lasers are available, have been used in open path configurations, i.e., measuring the attenuation along the path from the device to the surface and back. They are commercially available as miniaturized, battery-operated devices with size below 10 cm × 10 cm × 20 cm and weight below 700 g (including the battery) (ICI, 2022; Tokio Gas Engineering Solutions, 2022). Path lengths up to 30 m are possible (and can be extended up to 100 m using a dedicated reflector on the surface), and methane concentration measurements can be performed with a resolution of 1 ppm ⋅ m. Such devices can be mounted on drones, and have already been successfully used for leak detection with a detection limit of 0.9 L ${min}^{-1}$ (Golston et al., 2018). Some applications suggest using a gimbal to disentangle the observation path from the flight geometry (Bretschneider and Shetti, 2014). Care should be taken when using open-path sensors reflecting (or back scattering) off ground surface types with differing albedos (e.g., bare earth vs. open water) or with thick vegetation. Methane concentrations can also be derived from thermal imagery, and cameras are sometimes stabilized for drone applications (Nooralishahi et al., 2021). As emissions from pipeline leaks will enter the atmosphere either at or near ground level, and the plume is likely to remain close to ground (at least close to the emission source location), open path sensors may be the most appropriate approach for finding and locating emissions rather than point concentration (*in situ*) measurements. Remote sensing also allows for a drone to be positioned away from the emission plume, reducing risks to people, objects, and other hazards.

### 4.2 *In situ* sensing

In case of *in situ* sampling with TDLAS, an optical cell has to be integrated into the sensor as well. The cell is usually a multi-pass cell with optical path length in the range of metres to kilometres. This path length may be considerably longer than the optical path lengths in case of open path sensors, thus achieving similar or greater sensitivity. The ABB-LGR Hoverguard operates in the near-infrared (1.65 μ m) region, has a weight of 3 kg and dimensions of 12 cm × 34 cm × 29.5 cm. It has been used on a drone to measure CH4 concentration with 1 Hz resolution and 0.9 ppb precision (1 σ at 1 Hz) for deriving emission rates of 150 kg hour-1 from a landfill in the United Kingdom (Yong et al., 2024). Morales et al. (2022) used a custom-built *in situ* optical sensor weighing 2.1 kg and with a cavity-enhanced pathlength of 10 m. The sensor achieved a precision (1 σ ) of 1.1 ppb at 1 Hz. High sensitivity can also be realized in the mid-infrared region, around 3.3 μ m, where methane absorption lines are up to three orders of magnitude stronger than in the 1.65 μ m region. A commercial realization of *in situ* TDLAS sensors is the Aeris MIRA Pico (Aerissensors, United States). It weighs 2.75 kg and has a size of 30 cm × 20 cm × 10 cm, and has already been used on a drone to measure ${CH}_{4}$ concentrations with 1 Hz resolution and 0.84 ppb precision to derive maps of hot spots over a sludge deposit with an area of 25 m × 30 m. A total ${CH}_{4}$ emission of 170 L ${min}^{-1}$ was estimated based on the measurements (Galfalk et al., 2021).

For specific research projects, sensors have been developed by research institutes, e.g., a mid-infrared TDLAS sensor with an open multi-pass cell, using wavelength modulation spectroscopy, weight of 1.6 kg, size of 25 cm × 16 cm × 18 cm. The sensor was mounted on a hexacopter and was used to measure vertical profiles of methane concentration with a precision of 10 ppb at 1 Hz measurement frequency (Golston et al., 2017). *In situ* sensors need to be placed within an emission plume to make measurements and therefore could increase the risk associated with flying drones close to people, objects, and other hazards.

## 5 Operational aspects

### 5.1 Regulatory requirements for drone surveys

In Europe, since 2021, drones can be operated in the different categories “open”, “specific”, and “certified” depending on the potential risk associated with the mission (European Commission, 2019).

The category “open” has the lowest risk, and requires the lowest level of robustness of the drone technology. For drone operations in the category “open”, no flight permissions from the authorities are required. The main restrictions are.

• distance to uninvolved persons at least 30 m or the flight altitude (1:1 rule)

• flight altitude below 120 m

• maximum total mass of drone of 25 kg including payload

• flight in visual line of sight (VLOS) of the operator

In Germany, the last restriction (VLOS) is defined by the regulations NfL 2022-1-2554 and means that flights are performed within a radius depending on the size of the drone (Attitude Line Of Sight - ALOS) and the weather conditions to detect other aircraft (Detection Line Of Sight - DLOS). ALOS is calculated with the Maximum Characteristic Dimension (CD) in meter for rotary drones

ALOS=327⋅CD+20

and for fixed-wing drones

ALOS=490⋅CD+30.

DLOS is calculated by

DLOS=0.3⋅GroundVisibility.

The result is a maximum radius of 1.5 km. VLOS strongly limits surveys of long distances, like along transmission pipelines.

Drone flight operations which are not defined with in the “open” category, e.g., flights beyond visual line of sight (BVLOS), have to be categorized to the “specific” category. In the category “specific”, a flight permission from the responsible aviation authority of the home location of a company or institution is necessary. To obtain such a permission, the operator has to determine the risk of this particular mission at the specific location. The specific operation risk assessment (SORA) includes a ground risk and air risk evaluation based on the size and weight of the drone. Mitigation measures like strategic flight path planing, operational handling or technical solutions can reduce the risks. Based on the final risk a specific assurance integrity level (SAIL) is defined. Six levels are defined and correlate with an integrity from $1^{-2}$ for SAIL I to $1^{-7}$ for SAIL VI. That means in $1^{n}$ cases a lost of control of the system is acceptable. With the increase of SAIL, requirements for the whole flight operations (technology, operation, maintenance, training) increase and are directly linked to the expense and cost of operation. To reduce the expense of the permission process two shortcuts are forseen. First, standard scenarios (STS) that are limited to the use of classified drones, but currently no STS for pipeline inspection is defined. The second approach is the predefined risk assessment (PDRA). Here, the PDRA-G03 for line inspection is defined. It includes operation BVLOS, lower than 30 m above ground, with a characteristic drone dimension lower than 3 m and a direct control data link connection (EASA, 2024).

The third category “certified” applies similar standards as for manned aircraft with full certification process, which is a costly and lengthy process, in contrast to the flexible drone technology. It focuses on large drones or drones carrying humans, like urban air mobility vehicles.

### 5.2 Routine survey

Routine surveys are planned in advance and cover certain parts of pipelines for regular monitoring. The aerial perspective helps to get a quick overview of the situation, in particular in combination with live transmission of camera images. Fast monitoring is possible compared to ground-based surveys. Compared to manned airborne surveys, a high degree of automation is possible with drones, e.g., precise regular patterns at predefined and low altitude. The flight trajectory can be along the pipeline, taking into account the displacement of potential leaks by the wind, transects across the pipeline on small scales, or scanning patterns by sensor orientation (Bretschneider and Shetti, 2014). Less environmental impact will be created compared to ground-based and manned aerial missions, if electrical propulsion is used. Operation beyond visual line of sight allows efficiently covering larger transects.

For this kind of mission, different concepts are suitable: If frequent automated inspections are required, it could be feasible to place different automated multicopter systems along the pipeline. Either regularly or on demand, they ascend from their docking station, perform the monitoring of a certain part of the pipeline, transfer real-time images and data to a central control facility, and return to their weather-proof station. The concept is cost-intensive due to the requirement of having multiple multicopter systems equipped with sensors available, and the installation of infrastructure along the pipeline. The concept is technologically feasible with a high degree of automation, and the missions are comparable to automated gathering of weather data with drones at specific locations, which is already done at a routine base for several cases (Leuenberger et al., 2020).

For covering larger distances, fixed wing or tilt wing systems are suitable. The typical flight time for electric propulsion is much higher compared to multicopters, as fixed wing systems are much more energy efficient, and can be made of lightweight structures. Therefore, distances in the range of 50–100 km or beyond can be covered without the need of recharging batteries (Schön et al., 2022). Here, the sensor response time has to be fast enough to provide sufficiently high spatial resolution for leak detection. In particular for small leaks and unfavourable environmental conditions (high wind), this poses a challenge for sensor detection limits (see Sect. 2.2).

In both cases a dedicated risk analysis and safety concept are required, to make sure that no damage can be done to uninvolved persons, infrastructure or the environment. The lighter the overall system, the less damage can be expected in case of an accident. With reasonable cost, drone-base automated monitoring is only possible for gathering and transmission pipelines in open terrain and away from settlements.

### 5.3 On demand missions

On demand missions are performed on short notice for specific and typically small areas. The deployment of drones has certain advantages for leak detection in this context: The aerial perspective and real-time data transfer provide a quick overview of the situation, with added value of camera images. Local deployment, in particular within line of sight, can be done flexibly, and under certain conditions, fulfilling the requirements of the open category, even without the need of a flight permission. In this case only short operation time is needed, which can be done with light multicopter platforms. This kind of mission requires a ground crew in the vicinity of the area of interest, who surveys the mission and interprets the results based on real-time data. Altogether, the mission can be performed with high flexibility, a relatively simple multicopter system with easy handling, and without effort for regulations.

For this approach a suitable drone is a multicopter, in combination with a sensor of small size and weight, and sufficient accuracy.

## 6 Experiences from a methane release experiment

**FIGURE 5:**
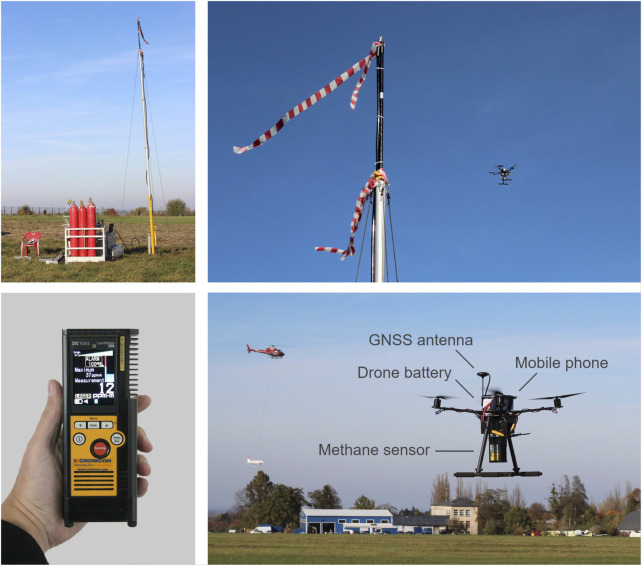
Setup of release experiment: Methane released from a nozzle at 7.2 m above ground (top-left). The drone flew a pre-designed pattern in the proximity of the release nozzle (top-right). The handheld methane detector *Laser Methane mini* (bottom-left) used as a sensor on the drone SToR ${CH}_{4}$ (bottom-right). The laser is oriented downwards and measures the methane concentration below the drone times the distance to the ground surface (in ppm ⋅ m). For recording, the measurements are transferred via Bluetooth to a mobile phone on board the drone. The helicopter and the slingload HELiPOD (Pätzold et al., 2023) (visible in the background) also participated in the release experiment.

To gain experience with the capabilities of a small drone and a lightweight sensor for leak detection, the SToR ${CH}_{4}$ drone system was developed. Equipped with a remote sensing methane detector, it was deployed during a release experiment organized by the German Aerospace Center at the airport Bielsko-Biała (49.8024°N, 18.9999°E), Poland, on 17 and 18 October 2022. The wind speed was below 5 m $s^{-1}$ from South-West with light gusts (see Table 7), and the day was mostly sunny with few cirrus clouds. During the release experiment, a controlled flow of methane was released from a nozzle at 7.2 m above ground (Figure 5). On 17 October 2022, the background methane concentration was around 1.95 ppm, and on 18 October, the background concentration was around 2 ppm. The ${CH}_{4}$ release rate was set to 500 L ${min}^{-1}$ (measured by a mass flow controller of Bronkhorst, indicated as nominal conditions). To identify detection limits, the emission was reduced later to ${CH}_{4}$ flow rates of 100 L ${min}^{-1}$ , 50 L ${min}^{-1}$ and 25 L ${min}^{-1}$ , which is in the order of magnitude of large pipeline leaks (see Sect. 1). The release rates were one or two orders of magnitude larger than for other release experiments, e.g., Li et al. (2020) performed releases with a flow rate of about 5 L ${min}^{-1}$ , which is similar to the expected dimension of leaks from gathering and distribution pipelines. Another study used small leak rates up to approximately 6 L ${min}^{-1}$ and larger rates up to around 60 L ${min}^{-1}$ (Ravikumar et al., 2019).

**TABLE 7:** Overview of the flights performed during the release experiment with the flight time, the respective ${CH}_{4}$ release rates, altitude and speed of the drone, the flight pattern flown and the number of overflights. Flights 6 and 9 were flown manually at variable speeds and trajectories. Flight 1 to Flight 3 were performed on 17 October 2022, Flight 4 to Flight 9 on 18 October 2022. The flights analysed in this study are highlighted in bold letters.

Flight No	Flight Time [UTC]	Release Rate [l ${min}^{-1}$ ]	Altitude [m]	Drone Speed [m $s^{-1}$ ]	Flight Pattern	Overflights
1	12:37-12:44	500	20	1.5	funnel-shaped	10
2	13:03-13:09	500	20	1.5	funnel-shaped	10
3	**13:23–13:30**	**500**	**20**	**1.5**	**funnel-shaped**	**10**
4	09:38-09:50	500	20	1.0	linear	12
5	10:03-10:16	500	20	1.0	linear	12
6	13:52-14:08	100	20	variable	variable	multiple
7	14:37-14:41	50	20	2.0	funnel-shaped	7
8	**14:53–14:59**	**50**	**20**	**1.0**	**funnel-shaped**	7
9	15:19-15:26	25	15	variable	variable	multiple

### 6.1 Methane detector

For the release experiment the handheld methane detector *Laser Methane mini* (Kimbrel et al., 2019; Tokio Gas Engineering Solutions, 2022) was used as a sensor. In the setup of Bretschneider and Shetti (2014), it was suggested to deploy two of the Laser Methane Mini systems on a gimbal in scanning mode. The 600 g TDLAS-based sensor measures the methane concentration times the distance between itself and a reflective surface in ppm ⋅ m. According to the manufacturer, the sensor is able to measure methane concentrations from 1 to 50,000 ppm ⋅ m with an accuracy of ± 10% in the range of 100 − 1,000 ppm ⋅ m. The measuring distance is given as 0.5 m–30 m and the measurement frequency is 10 Hz. The measured methane concentrations are shown on the instrument’s display (Figure 5, bottom-left). For recording, the measurements can be transferred via Bluetooth to a mobile phone or a tablet computer running the dedicated software *GasViewer* that is provided by the manufacturer (Tokio Gas Engineering, 2022).

### 6.2 Drone configuration

For the SToR ${CH}_{4}$ drone system, the commercially available Holybro (Hong Kong, China) S500 drone was modified and partially rebuilt to accommodate the *Laser Methane mini*. In particular, the landing skids were extended so that the downward-facing methane sensor does not touch the ground when the copter lands. To compensate for the mass of the low mounted methane sensor, the copter battery and the mobile phone for data recording were placed on top of the copter, ensuring an acceptable location of the center of gravity for the entire system. The complete setup of the SToR ${CH}_{4}$ drone is shown in Figure 5 (bottom-right).

### 6.3 Flight patterns and measurements

For sensing the methane plume, the drone flew a pre-designed pattern downstream of the release nozzle. Two different flight patterns were designed for the release experiment: One was repeated linear flights across the expected plume, which is perpendicular to the wind direction. The second was a funnel-shaped flight pattern, with meander transects of increasing length with distance perpendicular to the main wind direction. The flight legs were separated around 5 m. Flights were performed at horizontal distances up to 60 m from the release site. At any time, the drone was flying well above the expected plume at an altitude of 15 m or 20 m, within the range limit given by the manufacturer.

During the experiment a total of 9 flights were performed with the SToR ${CH}_{4}$ drone. The flight duration varied between 4 min and 16 min. The release rates and flight conditions, such as altitude and speed of the drone, the flight pattern and the number of overflights for every flight are summarized in Table 7.

**FIGURE 6:**
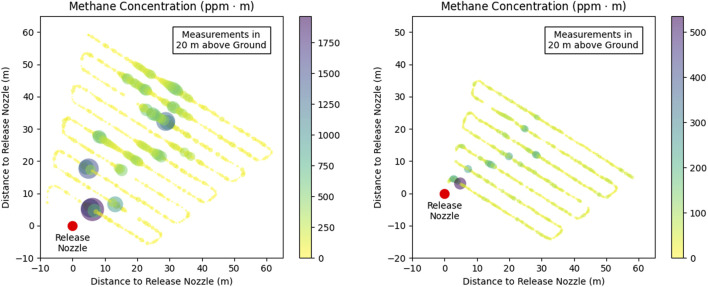
Flight patterns and observed column methane concentrations along the flight track for Flight 3 (release rate 500 L ${min}^{-1}$ ) and Flight 8 (release rate 50 L ${min}^{-1}$ ). The location of the release nozzle is indicated by the red dot. The concentration in ppm ⋅ m is provided at a temporal resolution of 2 Hz, in colours (note the different scales for the two flights), as provided by the instrument (internally averaged over 5 measurement points), and illustrated by the size of the dots. Missing data are omitted.

Figure 6 shows the observed methane concentration along the flight path and the variability over the measurement time period of several minutes. The methane plume is evident from an enhanced concentration for each transect, with generally decreasing concentrations with distance. This is expected due to dispersion effects (see Figure 4). Also for the lower release rate, the methane plume can be identified for each flight leg.

The horizontal distribution of the methane plume suggests a variability of the wind direction between 211° and 254° for Flight 3 and between 204° and 250° for Flight 8 within the flight duration of 7 min and 6 min, respectively. This variability strongly influences the measured concentrations, and sometimes the plume is distributed over larger sectors. Parallel measurements to Flight 3 were obtained with the helicopter borne meteorological sonde HELiPOD (Pätzold et al., 2023), which measured an averaged wind speed of 5.7 m $s^{-1}\;\pm$ 0.8 m $s^{-1}$ at an altitude between 21 m and 50 m and a wind direction of 225° ± 8° at a distance of approximately 330 m downstream of the release experiment. Therefore, a wind speed of around 4.5 m $s^{-1}$ is assumed for the altitude covered by the drone flights. Only data points with a ${CH}_{4}$ concentration exceeding 2 ppm were taken into account.

The maximum path integrated concentrations were around 1,500–1700 ppm ⋅ m for Flight 3, which is in the same order of magnitude as calculated for the most similar Scenario 9 (1,276 ppm ⋅ m). Also for Flight 8, the maximum concentration in range of 500 ppm ⋅ m is higher than calculated for Scenario 6, with a maximum concentration of 184.5 ppm ⋅ m.

### 6.4 Approximate determination of release rates

**FIGURE 7:**
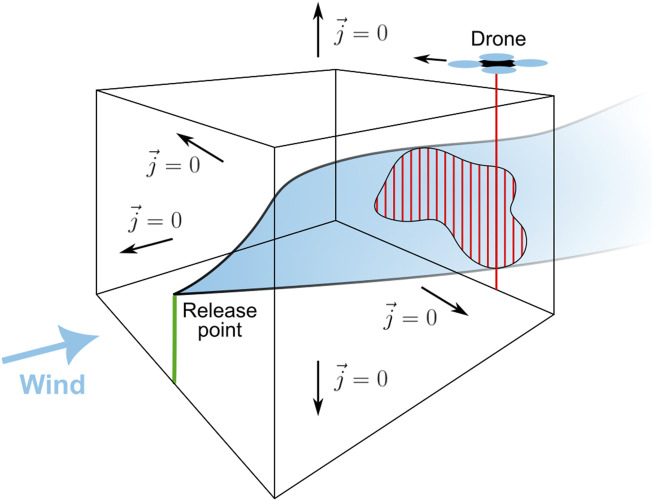
Illustration of the release rate estimation based on a path-integrating sensor on the drone.

According to the Gauss’s theorem, the surface integral of a vector field over a closed surface (net flux through the surface) is equal to the volume integral of the divergence over the space enclosed by that surface (sum of all sources and sinks). The theorem can easily be applied to the local conditions during the release experiment: As illustrated in Figure 7, the methane flux $\vec{j}$ through the surface of the cuboid shown in the illustration must be equal to the release rate of methane from the release nozzle, since this is the only methane source within the cuboid. Assuming uniform wind speed and direction, it is obvious that none of the released methane will flow through the upwind surface of the cuboid. The flux through the bottom surface (ground) is naturally zero, and if the upper and lateral surfaces are located well above and next to the expected methane plume, the fluxes through theses surfaces will also be zero. Under these conditions, all of the released methane will leave the cuboid via the downwind surface. In Figure 7, the area where the methane plume intersects the downwind surface is marked in red.

To calculate an approximate methane flux through the downwind surface, the drone with the TDLAS-based methane sensor flies across the plume as indicated in the illustration. The sensor points downwards and provides the methane column concentration in ppm ⋅ m. When integrating the column concentration measurements over the entire flight path across the plume, a surface concentration can be derived. The total flux through the surface and therefore the release rate can eventually be estimated by multiplying the surface concentration by the average wind speed during the measurement. If the flight path is not oriented perpendicular to the average wind direction, the result must be multiplied by the sine of the angle between wind direction and flight path, since only the outgoing flux component perpendicular to the surface is relevant for the calculation.

**FIGURE 8:**
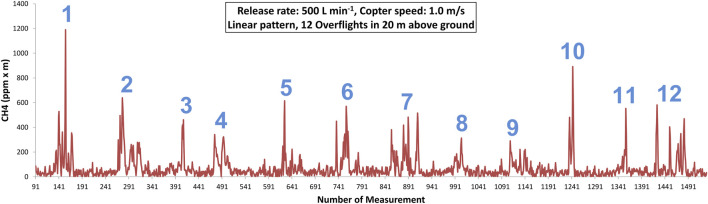
Column methane concentrations observed by the SToR ${CH}_{4}$ drone during Flight 4 (release rate 500 L ${min}^{-1}$ , linear flight pattern). The drone performed 12 overflights in 20 m above ground and flew at a speed of 1 m $s^{-1}$ . The number of each overflight is marked in blue.

During Flight 4 of the experiment, the drone flew back and forth along a linear pattern at a speed of 1 m $s^{-1}$ and an altitude of 20 m above ground. Methane was released at 500 L ${min}^{-1}$ and the wind was blowing almost perpendicular to the flight path at an approximate wind speed of 4.5 m $s^{-1}$ . A total of 12 overflights were performed during Flight 4. The consecutive column methane concentrations measured during Flight 4 are shown in Figure 8. Individual overflights of the methane plume are clearly visible and marked with the respective numbers. The background methane column concentration of 32.7 ppm ⋅ m was calculated by averaging all measurements adjacent to the plume. The measurements where the drone flew above the plume were then averaged for each overflight. To just calculate the amount of methane released from the nozzle, the background concentration has to be subtracted from this average. A rough estimate of the release rate can be derived by multiplying the resulting value with the observed width of the plume and the wind speed. After some unit conversions, the release rate in [L ${min}^{-1}$ ] can eventually be obtained. Table 8 summarizes the number of measurements that fall into the plume section of each overflight, the observed width of the plume and the calculated release rate. During some overflights, small gaps of one or two measurements occurred in the recording of the Laser Methane mini. The influence on the calculated average and therefore the release rate, however, is rather small. The average release rate across all overflights is 640 L min^−1^ with a standard deviation of 261 L min^−1^. Compared to the actual methane release rate of 500 L ${min}^{-1}$ , the calculated average rate is 28% too high, but could serve as a first estimate. The rates calculated for overflights 1 and 2 are far too high. This could have been caused by wind gusts at the beginning of Flight 4. Due to the lack of high-resolution wind data from the measuring site during the flight, this can no longer be determined.

**TABLE 8:** Release rates and the observed width of the plume calculated for each overflight during Flight 4.

Overflight No.	Number of Measurements	Plume width [m]	Release rate [L ${min}^{-1}$ ]
1	57	29.5	906
2	74	40.5	1,232
3	65	32.4	440
4	48	24.4	629
5	60	30.6	425
6	67	33.9	714
7	79	39.6	903
8	56	31.6	417
9	63	32.4	409
10	23	11.4	489
11	76	38.5	342
12	70	35.6	775

The quality of such estimates strongly depends on the variability in wind speed and direction as well as the strength of a possible wind sheer that has not been considered so far, as also shown by Wolff et al. (2021). The assumption that all of the released methane passes through the surface in which the drone measurements take place may also not be valid for strongly varying wind directions. Another aspect is the mixture process, that can vary with turbulence and wind speed. These effects as well as the impact of the changing drone attitude and resulting angle of measurement beam and the sensor characteristic itself will be investigated and published in another paper.

## 7 Potential drone-based leak detection scenarios

In the following, three different scenarios are described how drones may be used for pipeline leakage monitoring. They are very different in complexity and cost, which may be important factors for industrial applications in addition to the accuracy of the measurements (Barchyn et al., 2019).

### 7.1 Scenario 1: Drone-based support of helicopter missions by trained personnel in the near field

For on demand leakage detection in a relatively small pipeline section (up to few km), it is possible to enhance helicopter missions by drone measurements with an experienced drone operator as described in Sect. 5.3. It is technically feasible, assuming that a suitable sensor is available. Highly automated operations are not required, but instead, the mission is controlled by trained personnel. The overall efficiency strongly depends on the endurance of the drone, the requirements concerning sensitivity, and the terrain for VLOS conditions. However, cost is associated with qualification and training of the operator or crew, and maintenance of the drone and sensors. If the mission is performed in the “open” category, the condition of line of sight leads to frequent changes of location of the operator. Smaller copters generally have a lower endurance and require frequent change of batteries. In comparison with helicopter missions, the payload capacity is much smaller, therefore it is not possible to use highly accurate sensors with enhanced weight. An open question is the minimum detection level of the sensors, in order to identify also leaks of small size and under unfavourable ambient conditions, as explained in Sect. 2.1. Nevertheless, this application can help to identify and confirm medium sized leaks near gathering stations or in a leak suspected areas but not for routine pipeline surveys. Typical performed trajectories are crosswind patterns, like applied exemplarily during the release experiment (see Sect. 6).

### 7.2 Scenario 2: replacement of helicopter missions by BVLOS flights with fixed-wing or tilt rotor in the far field ( < 100 km)

For on demand or regular monitoring of pipeline sections of several km to around 100 km, it is possible to replace helicopter missions by drones. For such distances, it is an advantage to deploy fixed-wing or tilt wing systems with larger endurance, which cover longer transects of pipelines. With electric propulsion, there are less emissions than for helicopter operation. However, for BVLOS flights, the requirements concerning technical performance, in particular robustness, redundancy and level of safety, are much higher compared to VLOS flights. The operating company has to perform a risk assessment and fulfill criteria for the level of robustness in different categories to obtain a flight permission. This financial, technical and operational effort is only justified for regular use of the systems, like for a service provider specialized in BVLOS operations. Also for BVLOS operations, the sensor accuracy and resolution is critical in order not to miss leakages, and the flight planning should take into account wind speed and wind direction for adapting the distance and altitude, as exemplarily indicated in Table 2, 3. Typical trajectories will be along the pipeline within a lateral deviation ( ± 2 m) in wind direction. If the mission is executed in return-to-launch mode the trajectory can be shifted between the first and the second leg to cover more areas and react on local chances in wind direction and speed. Overall, the complexity of the missions is high, and cost depends on the organisation of the service provider, covering training of the crew, maintenance of the systems, and efforts to obtain the flight permission.

### 7.3 Scenario 3: Routine automated survey flights for specific small pipeline sections

For frequent and regular monitoring, it is possible to deploy highly automated survey drones, each responsible for a certain section or critical parts of the pipeline. This enables to observe and react to gradual changes with time, like the deterioration of leakages. In this case the drones belong to the pipeline, and need storage shelters for automatic charging. They are released regularly to perform the task, transmit live data to the central control station, and return to their home base. The permission for well-determined operation areas and missions are easier to get than for flexible deployment as described in Scenario 2. The flight pattern can be optimised to take into account environmental conditions, like wind direction. However, the effort is large, as infrastructure for the charging stations has to be set up and maintained, the drones require a high level of safety for automated missions, and have to be checked regularly, as they are exposed to ambient weather conditions.

Given the focus on the monitoring of long linear assets, fixed wing drones clearly have an advantage over rotary wing drones in terms of energy efficiency (energy required per meter or km of pipeline surveyed). However, because of the flight profiles of fixed wings (minimum speed and altitude compared to rotary wing drones) the sensors they use to detect leaks need to be appropriate—relatively high frequency and downward pointing open path instruments are likely most suited. A suitable solution could be the deployment of rotary wing drones as sensing devices once there is an indication of methane emissions in a defined location.

## 8 Conclusion

The modeling results can be used to derive sensor requirements. The Ventjet model from Sect. 2.2 shows the plume distribution for representative wind situations, mixture processes and release rates. The dispersion model (see Sect. 2.3) shows results for the minimum reference leaks of 2.5 L ${min}^{-}1$ for helicopter surveys. With the small plume spread ( < 10 m) and less intensity ( < 10 ppm ⋅ m) the sensor requirement of detection threshold of 1–2 ppm ⋅ m and measurement requirement of ± 2 m lateral pipeline deviation is in line with the technical guideline GVGW-G501 (DVGW, 2012). In addition to the accuracy requirements the sensors need a minimum measurement rate ≥ 10 Hz in combination with a drone performance of flying as slow as possible to identify the leak plume and its distribution but meeting the operational and cost requirements. The distribution model illustrates that the maximum concentration is reduced up to a factor of 75% for increasing the wind speed from 1.5 m $s^{-1}$ to 4.5 m $s^{-1}$ . This results in the limitation of operational conditions measuring only during wind speed conditions less than 5 m $s^{-1}$ to reduce the true-negative-rate (not detected leaks).

The release experiment in Sect. 6 underlines the requirements derived from the distribution models. With the release rate of huge leaks of 500 L ${min}^{-1}$ and medium leaks with 50 L ${min}^{-1}$ the order of magnitude that is expected from the distribution model in Sect. 2.3 can be confirmed. But these results also show that the inhomogeneously measured plume distribution includes a not well-fitted mixture process in the modeling and errors in the measurements. The errors in over- or underestimation of the measurements can be traced back to lower sensor accuracy and the combination of a small sensor detection rate, respectively small drone airspeed, in combination with changes in wind speed, wind deviations and turbulence during the measurement pattern. This is also visible in the overestimated calculated release rate of 28% in average. This is in agreement with recommendations to perform airborne measurements for emission quantification during time periods of low turbulence (Wolff et al., 2021).

The *in situ* sensors shown in Sect. 4 already meet the requirements for detecting small leaks with an accuracy of 0.01 ppm (Golston et al., 2017). The disadvantage is that these sensors have to fly through the plume. In case of a minimum leak 2.5 L ${min}^{-1}$ at low wind speed of 1 m $s^{-1}$ the plume concentration of 1 ppm is less than 3 m AGL (see Vinjet model Sect. 2.2). As the measurement rate is only 1 Hz, this sensor can be used for application of Scenario 1 - near-field survey (see Sect. 7.1) but not for long-range surveys.

In summary, pipeline leak detection by drones is feasible from a technological point of view of the drone systems. In terms of cost efficiency, which is required for industrial deployment, the application of drones strongly depends on the individual scenario and conditions of operations. Currently local observations of leaks within VLOS or near-BVLOS is the state of the art, but limited by the sensor technology - in particular the accuracy and measurement frequency. Replacing manned helicopter observations for large parts of the 200,000 km pipeline grid is currently limited by the sensor technology and costs for system integrity and operational handling resulting from regulations. It can be expected that with the growing market of drones and applications (SESAR JU, 2016), more specialised companies will be able to offer drone-based surveys for different scenarios.

## Data Availability

The raw data supporting the conclusions of this article will be made available by the authors, without undue reservation.
