# Supplementary figures and images for: Concepts for drone based pipeline leak detection
Source: Front Robot AI. 2024 Aug 15;11:1426206. doi: 10.3389/frobt.2024.1426206 (PMC11357903; doi:10.3389/frobt.2024.1426206)

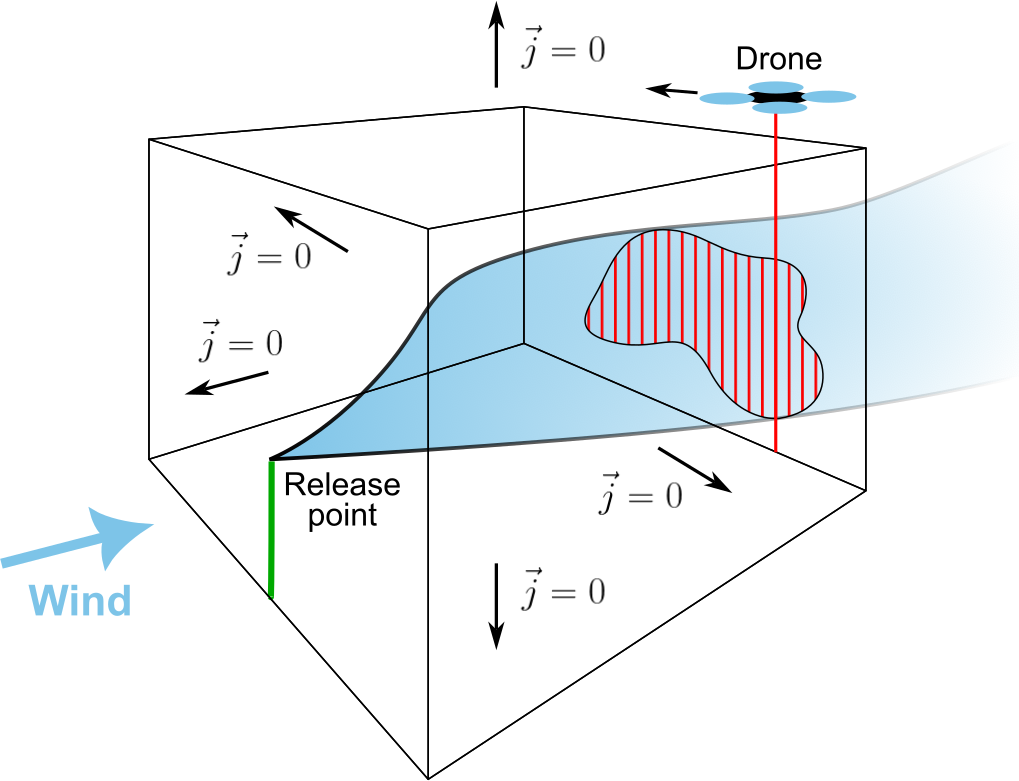

Supplement: Supplementary file 1 [file Image4.png]

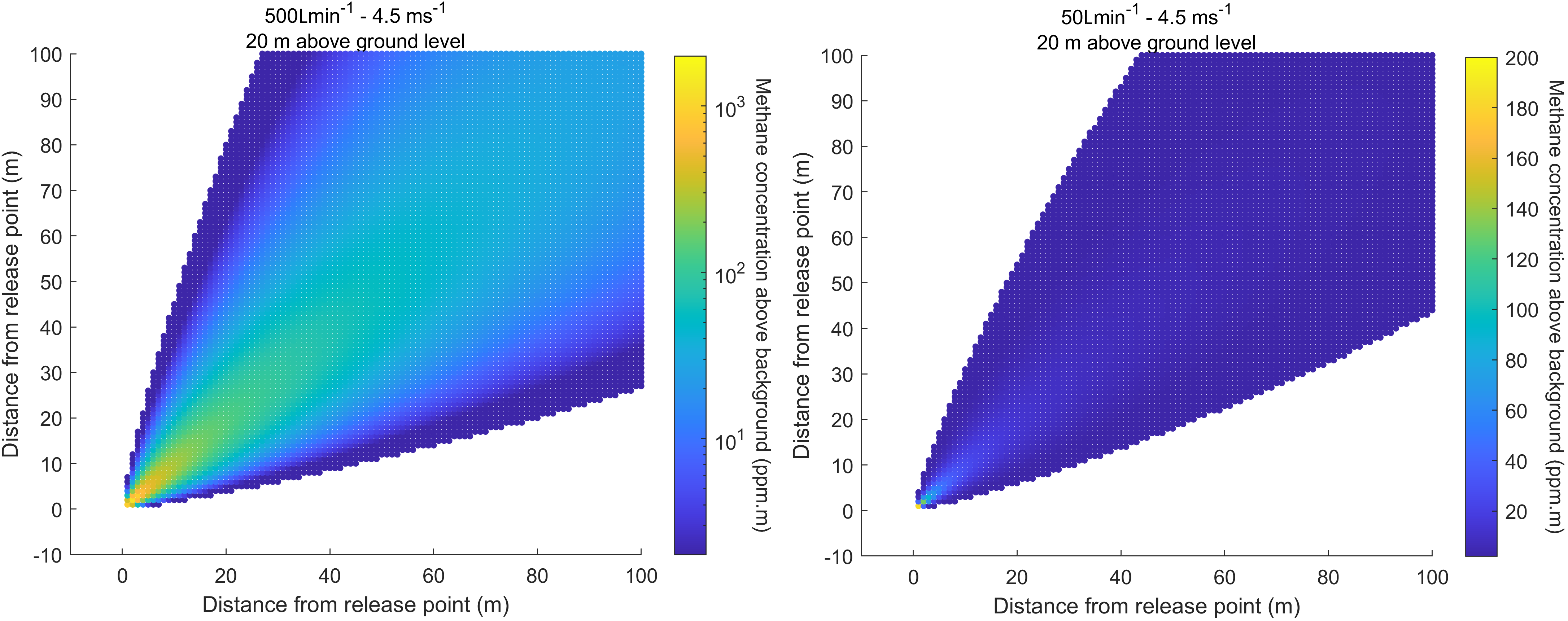

Supplement: Supplementary file 2 [file Image2.png]

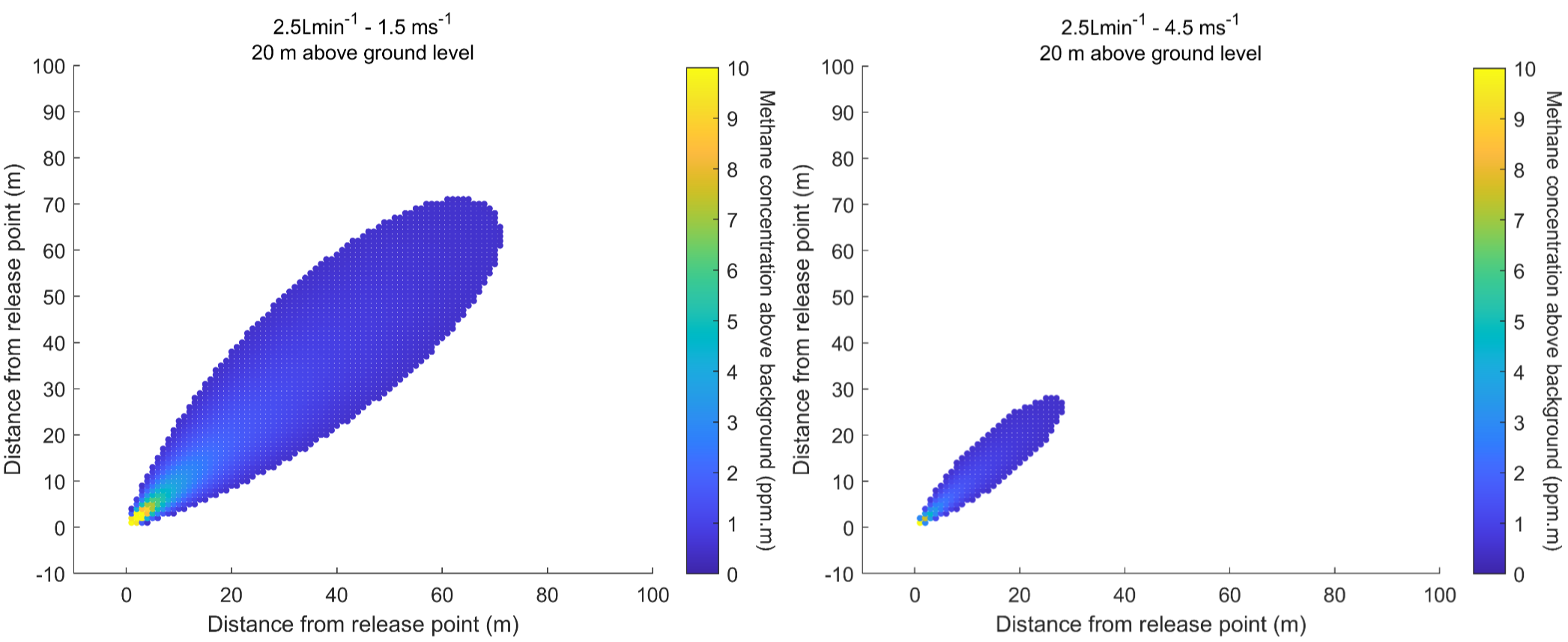

Supplement: Supplementary file 3 [file Image1.png]

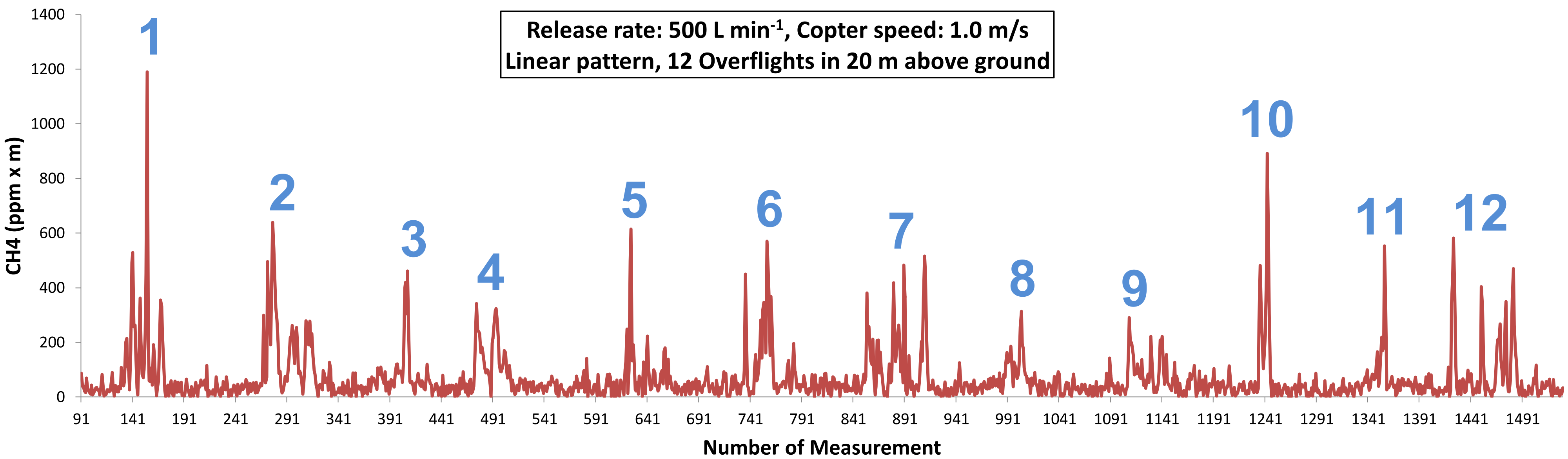

Supplement: Supplementary file 4 [file Image3.png]
